# Association between Exposure to Ambient Air Particulates and Metabolic Syndrome Components in a Saudi Arabian Population

**DOI:** 10.3390/ijerph15010027

**Published:** 2017-12-25

**Authors:** Magdy Shamy, Mansour Alghamdi, Mamdouh I. Khoder, Abdullah M. Mohorjy, Alser A. Alkhatim, Abdulrahman K. Alkhalaf, Jason Brocato, Lung Chi Chen, George D. Thurston, Chris C. Lim, Max Costa

**Affiliations:** 1 Department of Environmental Sciences, Faculty of Meteorology, Environment and Arid Land Agriculture, King Abdulaziz University, Jeddah 21589, Saudi Arabia; mans99@gmail.com (M.A.); khoder_55@yahoo.com (M.I.K.); abuenas@hotmail.com (A.A.A.); 2 Center of Excellence in Environmental Studies, King Abdulaziz University, Jeddah 21589, Saudi Arabia; 3 Department of Civil Engineering, Faculty of Engineering, King Abdulaziz University, Jeddah 21589, Saudi Arabia; amohorjy@kau.edu.sa; 4 Department of Meteorology, Faculty of Meteorology, Environment and Arid Land Agriculture, King Abdulaziz University, Jeddah 21589, Saudi Arabia; akhalaf@kau.edu.sa; 5 Department of Environmental Medicine, New York University School of Medicine, New York, NY 10987, USA; jab824@nyu.edu (J.B.); lung-chi.chen@nyumc.org (L.C.C.); george.thurston@nyumc.org (G.D.T.); cchaeha@gmail.com (C.C.L.); max.costa@nyumc.org (M.C.)

**Keywords:** air pollution, particulate matter, metabolic syndrome, hypertension, diabetes, hyperglycemia

## Abstract

Recent epidemiological evidence suggests that exposure to particulates may be a factor in the etiology of metabolic syndrome (MetS). In this novel study, we investigated the relationship between particulate levels and prevalence of MetS component abnormalities (hypertension, hyperglycemia, obesity) in a recruited cohort (N = 2025) in Jeddah, Saudi Arabia. We observed significant associations between a 10 μg/m^3^ increase in PM_2.5_ and increased risks for MetS (Risk Ratio (RR): 1.12; 95% Confidence Interval (CI): 1.06–1.19), hyperglycemia (RR: 1.08; 95% CI: 1.03–1.14), and hypertension (RR: 1.09; 95% CI: 1.04–1.14). PM_2.5_ from soil/road dust was found to be associated with hyperglycemia (RR: 1.12; 95% CI: 1.06–1.19) and hypertension (RR: 1.11; 95% CI: 1.05–1.18), while PM_2.5_ from traffic was associated with hyperglycemia (RR: 1.33; 95% CI: 1.05–1.71). We did not observe any health associations with source-specific mass exposures. Our findings suggest that exposure to specific elemental components of PM_2.5_, especially Ni, may contribute to the development of cardiometabolic disorders.

## 1. Introduction

Jeddah, a major metropolitan city of nearly 3.4 million residents, serves as a commercial, economic and cultural hub of Saudi Arabia and the Middle East [1]. Rapid population growth and expansion of the city have resulted in deteriorating air quality, raising concerns about the potential health effects.

Ambient air pollution is linked with adverse health effects, with numerous studies establishing causal associations between exposure to particulate matter (PM) and elevated risk for cardiovascular morbidity and mortality [2]. Preliminary epidemiological and mechanistic evidence have suggested that ambient air pollution exposure is also involved in the development of cardiometabolic disorders [3]. Metabolic syndrome (MetS) is a cluster of risk factors, with the Adult Treatment Panel III formally defining MetS as having at least three of the five out of hypertension, abdominal obesity, elevated fasting glucose, high serum triglycerides and low circulating high-density lipoprotein [4]. MetS is a precursor of developing cardiovascular disease and type II diabetes mellitus, and the presence of component abnormalities of MetS may impart individuals with greater susceptibility to PM-related health effects [5]. The prevalence of MetS has rapidly risen in Saudi Arabia over the past two decades, with studies estimating prevalence in the country as high as 41% [6].

Past epidemiologic investigation of the association between air pollution and cardiometabolic disorders have shown mixed, but generally positive associations. For example, short [7], long [8], long and short [9], intermittent [10]. Exposure to PM with an aerodynamic diameter equal to or less than 2.5 µm (PM_2.5_) was found to contribute to increased incidence and/or prevalence of diabetes mellitus (DM) among U.S. adults. Short-term exposure to PM_2.5_ has been found to be associated with immediate elevation of blood pressure [11,12]. Long-term exposure has been associated with the development of hypertension [13]. Associations between air pollution and formally defined MetS have not been examined extensively to date, although recently a significant association between PM_10_ and prevalence of MetS, defined via multiple definitions, was reported in a cohort of Swiss adults [14].

In animal studies, exposure to PM_2.5_ increased blood glucose, adipose inflammation, and insulin resistance [15]. It impaired energy metabolism and increased inflammation in insulin responsive organs, resulting in imbalances in circulating leptin and adiponectin levels [16]. By triggering autonomic nervous system imbalance, PM_2.5_ exposure also promoted vasoconstriction and reduced insulin sensitivity [17]. PM_2.5_ impaired renal handling of excess sodium, inhibiting normal nocturnal reduction in blood [18].

As PM represents a complex mixture that is directly released from both geogenic and anthropogenic sources, or formed in the atmosphere from pollutant gases into secondary aerosols through chemical reactions, the chemical and physical characteristics, as well as the associated health effects, of the PM air pollution mixture are variable depending on its source(s), and this is thought to also influence PM toxicity [19]. Only a few studies have partitioned the health effects to source-related PM components [20,21,22], and none that we are aware of have been conducted to date in the Middle East.

In this innovative study, based upon our prior quantification of elemental components and source apportionment of PM with an aerodynamic diameter equal to or less than 10 µm in size (PM_10_) and PM_2.5_ in Jeddah [23], we investigated the epidemiological relationships of the previously estimated PM exposures from various sources in Jeddah with the prevalence of MetS in a local cohort, as well as with three component cardiometabolic abnormalities (hyperglycemia, hypertension, and obesity), within this recruited cohort.

## 2. Materials and Methods

### 2.1. Air Quality Measurements in Jeddah

**Figure 1:**
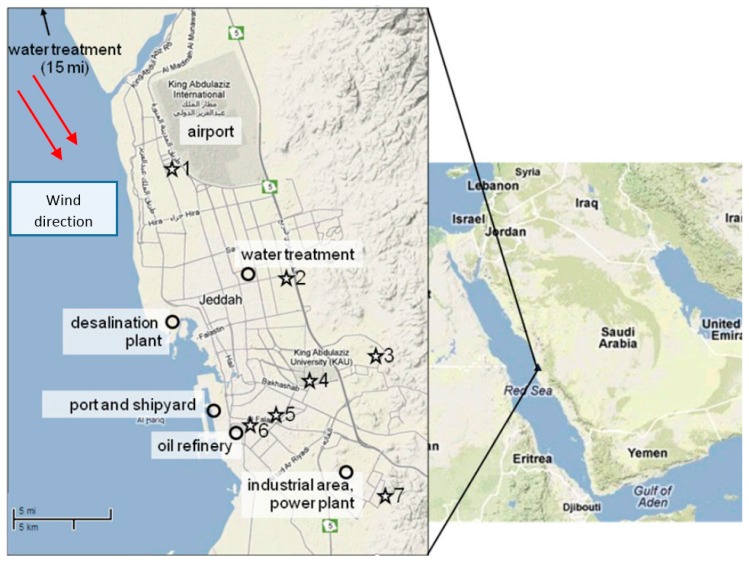
Locations of sampling sites (stars) and notable stationary industrial sources (circles) in Jeddah; 1 = Al-Muhammadiyah; 2 = Al-Rehab; 3 = Al-Rughama; 4 = University Campus; 5 = Al-Nuzlah Al Yamaneyyah; 6 = Pitrumin; 7 = Al-Alfiyyah.

The primary mobile source of pollution in Jeddah is traffic, with more than 1.4 million vehicles fueled mainly by unleaded gasoline and diesel oil, while major stationary sources in Jeddah include an oil refinery, a major seaport, a desalinization plant, a power generation plant, and several manufacturing industries (Figure 1). As elaborated in more details in Khodeir et al. [23], air samplers were installed at seven locations across Jeddah. Samples were collected every 24 h from midnight to midnight every other day, using PM_10_ and PM_2.5_ Harvard impactors [24] and calibrated vacuum pumps to draw air at a rate of 10.0 L/min onto Teflon filters (GelmanTeflo, 37 mm, 0.2 μm pore-size). The seven sampling sites were located throughout the city (Figure 1), and were selected based on varying traffic and/or population density: four in urban areas (University Campus, Al-Nuzlah, Pitrumin, and Al-Rughama), and three in residential areas (Al-Muhammadiyah, Al-Rehab and Al-Alfiyyah).

### 2.2. Source Identification

We applied the widely employed Absolute Principal Components Analysis (APCA) method developed by Thurston and Spengler [25] in order to identify major PM source categories in each size fraction. This provided positive indices of daily source impacts, upon which daily PM mass concentrations were regressed to achieve a source apportionment for application to this research. Estimates of mass contributed by the identified source categories for each of the locations were calculated using factor weights, and the regression models developed in a previous source apportionment study conducted in Jeddah [23]. For PM_2.5_, four source factors were identified based on the factor eigenvalues and factor source interpretability as follows: soil/road dust (loadings on Al, Ca, Cr, Fe, K, Mg, Mn, Si, Sr, Ti), residual oil (Ni, S, V), solid waste incineration (Cu, Zn), and traffic (Pb and S); for PM_10_, three factors were identified: soil/road dust, traffic, and solid waste incineration.

### 2.3. Study Population Recruitment, Exposure Assessment, and Health Measurements

Participants (N = 2686) were recruited from interviewing individuals at two mega-malls, one located in North Jeddah, and the other located in South Jeddah, from June 2011 to May 2012. Participants were enrolled by trained personnel based on their willingness to participate in the study, excluding only those having lived in the area of study for less than 15 years. Confidentiality was maintained in the completed questionnaires, and the identity of the participants remained anonymous. The questionnaire included information on age, type of residence, occupation, education, smoking habits, sleeping hours, physical activity, hours since the last meal, past history of diabetes, hypertension, and other chronic diseases, and weekly frequency of food intakes by type. Physical activity was defined as ‘yes’ if participants were involved in walking, swimming, running, and biking. After the interview, body weight and height were measured without shoes using electronic measuring scale. Body mass index (BMI) was calculated as weight in kilograms divided by height in m^2^ and obesity was defined as BMI > 30 for both men and women. Blood pressure was monitored in three measurements taken in 5-min intervals and averaged. Hypertension was defined as systolic blood pressure ≥140 mmHg and/or diastolic blood pressure ≥90 mmHg; those reporting use of anti-hypertensive drugs were considered as hypertensive regardless of their recorded blood pressure. Random blood sugar levels were measured and hyperglycemia was defined if the values were equal to or exceeded 126 mg/dL; those reporting use of diabetes control medications were considered as having diabetes regardless of recorded blood sugar levels. Presence of MetS was defined as having all three of the cardiometabolic abnormalities measured in the study: obesity, hypertension, and hyperglycemia [26]. Individual exposure to air pollution was linked to the subjects’ district of residence, using averaged values from the samplers that were installed at seven sites throughout Jeddah districts as described previously (Figure 1). An informed verbal consent was obtained from each participant, and the study proposal was approved by the King Abdulaziz University Research Ethics Committee (number 700-12).

### 2.4. Statistical Analyses

The associations between PM concentration levels and prevalence of MetS, as well as with individual components of MetS, were investigated using a generalized linear mixed effects model, adjusted for each individual’s age, sex, type of residence (house, apartment, villa), marriage status (currently married/no), occupation (unemployed, labor work, desk work), education level (<secondary, secondary, university and post graduate), physical activity (no/yes), diet (weekly consumption of fresh fruits and vegetables), current smoking status (currently smoking/no), and district location (as a random effect). Detailed cohort characteristics, by location of residence, are displayed in Table 1. Subjects with incomplete individual covariates data were excluded, the total number of subjects available for analysis was N = 2025. All statistical analyses were conducted in SAS (R) Studio 3.1 (SAS Institute Inc., Cary, NC, USA).

## 3. Results and Discussion

Summary characteristics of the seven monitoring sites are presented in Table 2. As seen in Table 2, the overall average concentrations of PM_2.5_ (38.6 μg/m^3^, SD = 21.4 μg/m^3^) and PM_10_ (85.1 μg/m^3^, SD = 30.30 μg/m^3^) measured in the present study period across the sampling sites greatly exceeded the recommended annual levels by the World Health Organization for PM_2.5_ (10 μg/m^3^) and PM_10_ (20 μg/m^3^) [27]. Highest concentrations for PM_2.5_ (73.2 μg/m^3^) and PM_10_ (141.3 μg/m^3^) were observed in the Al-Rughama, while lowest concentrations for PM_2.5_ (15.8 μg/m^3^) and PM_10_ (47.0 μg/m^3^) were observed in Al-Muhammadiyah. Al-Rughama is a suburban area characterized by intensive traffic, construction activities, small workshops and open burning of solid waste, while al-Muhammadiyah is a quiet typical residential area with light traffic.

Sulfur (3.39 μg/m^3^) was the dominant element found in PM_2.5_, representing 26.8% of total elemental mass, whereas silicon (10.4 μg/m^3^) was the dominant element found in PM_10_, representing 34.53% of total elemental mass. The elemental components profiles of each location generally reflected the environmental and physical characteristics of their respective surroundings; samples from locations closest to the non-developed lands east of the city—Al-Alfiyyah, Al-Rehab, and Al-Rughama—had elevated concentrations of crustal elements, such as Al, Ca, Mg, and Si. High concentrations of Ni, S, and V, markers of residual oil combustion were measured in Pitrumin, Al-Nuzlah and Al-Rughama, which are the sampling locations closest to the port and oil refinery. Al-Rehab and Al-Alfiyyah, closest to water treatment and industrial power plant areas, had high concentrations of Cu and Zn, elements commonly associated with open and/or industrial waste burning. Samples from Al-Rughama, Al-Alfiyahh, and University, which are located nearby the major highway, had elevated concentrations of Pb, a marker for traffic even after discontinued leaded gasoline use, due to accumulation in soil [28].

The degree of metal contamination in atmospheric PM_2.5_ and PM_10_ of the study areas can be assessed by comparing their measured concentrations with regulatory standards. Such an approach, however, cannot be adopted in the present study because standards are not available for the measured metals. An alternative approach would be to compare the heavy metal concentrations in the study area with the safe limits proposed by international agencies. World Health Organization (WHO) [27] standards for atmospheric Pb, Mn, Cr and Ni are 500, 150, 1100 and 0.38 ng/m^3^, while those reported by the Agency for Toxic Substances and Disease Registry (ATSDR) [29] are 1500, 500, 100 and 0.24 ng/m^3^ for the same metals in the same order. In the present study, only Ni levels in both PM_2.5_ and PM_10_ were many times higher than the proposed WHO and ATSDR standards, and Mn in PM_10_ at Al-Rughama was higher than WHO standards. The average concentrations of Pb, Cr and Mn were even lower than the WHO and ATSDR standards. Associations between ambient air nickel and cardiovascular disease have become a research issue in the new millennium [21,30,31,32]. At the molecular level and for Saudi Arabia specifically, Brocato et al. [33] reported that in vivo exposures to particulate matter collected from Jeddah or nickel chloride display similar dysregulation of metabolic syndrome genes.

Results from the statistical modeling are presented in Table 3. After adjusting for confounders, we found that PM_10_ was not associated with MetS (RR: 1.03; 95% CI: 0.95–1.13), hyperglycemia (RR: 0.98; 95% CI: 0.92–1.05), hypertension (RR: 1.06; 95% CI: 1.00–1.13), and BMI (RR: 0.95; 95% CI: 0.89–1.02). This is not in accordance with the results of Brocato et al. [34] who reported that acutely exposing mice to PM_10_ collected from Jeddah induced genes involved in inflammation, cholesterol and lipid metabolism, and atherosclerosis. On the other hand, we found that a 10 μg/m^3^ increase in PM_2.5_ was significantly associated with MetS prevalence (RR: 1.12; 95% CI: 1.06–1.19), hyperglycemia (RR: 1.08; 95% CI: 1.03–1.13) and hypertension (RR: 1.09; 95% CI: 1.01–1.18), but not with BMI (RR: 0.95; 95% CI: 0.91–1.00). This proves that, for chronic exposure, PM_10_ is less risky concerning cardio metabolic effects.

When the source of PM was studied again, none of the identified source mass estimates showed significance with neither MetS nor the specific endpoints for PM_10_. For PM_2.5_, soil/road dust mass was significantly associated with MetS (RR: 1.16; 95% CI: 1.08–1.25), hyperglycemia (RR: 1.12; 95% CI: 1.06–1.19), and hypertension (RR: 1.11; 95% CI: 1.05–1.18). This suggests that crustal elements as Al and Si originating from the surrounding deserts and partially generated as road dust may potentially be responsible for the cardiometabolic effects. Other investigations found that Al and Si induced markers of oxidative stress and inflammation in animals and humans [35,36,37], a major mechanism for the cardiovascular effects induced by particulate matter exposure [38,39]. Moreover, the deleterious role of Si in the development of chronic renal disease [40] might suggest its implication in hypertension. Also, a significant association (RR: 1.33; 95% CI: 1.05–1.71) between PM_2.5_ traffic source components (Pb, S) and hyperglycemia was observed. Although Pb exposure has been falling, it is likely that even very low levels of exposure are hazardous to health [41]. Pb exposure exerts its effect on cardiovascular diseases by increasing the risk of hypertension, and through its proinflammatory properties affecting all stages of atherogenesis [42]. The actual contribution of Pb exposure to diabetes risk in the general population is difficult to assess. However, numerous studies proved a strong association between low levels of lead exposure and markers of oxidative stress. Oxidative stress is known to promote the development of diabetes through multiple mechanisms. Inhibition of several key components of insulin signaling pathway resulting in insulin resistance, is one of them [43]. Our source-specific findings could thus potentially explain the inconsistent results from the aforementioned studies examining the relationships between DM/hypertension incidence and an index of overall air pollution (e.g., PM_2.5_ mass), in which some studies observed stronger associations with NO_2_, a proxy for traffic-related air pollution, than PM_2.5_ [44,45]. While a direct effect of NO_2_ cannot be ruled out, results from our study suggest that elemental components generated from traffic-related PM_2.5_ could potentially be responsible for the observed health effects, confirming that PM might be the major component of air pollution producing the most deleterious effects on human health [46].

The major strength of the present study is that, up to our knowledge, is the first epidemiological study investigating the relationship between source-specific elemental components of PM and metabolic syndrome. However, there are several limitations of this study. First, the retrospective and cross-sectional nature of the study design precludes establishing a definitive causal link between PM_2.5_ exposure and the outcomes considered here. Second, several elements, carbonaceous species (elemental and organic carbon), and gases that were either not measured, or not considered in our source apportionment, could potentially be causal predictors of health outcomes of interest. For example, due to missing data in the original APCA, some elements (As, Cd, Cl, Co, Ga, Se, Rb) were excluded in the subsequent analysis, thereby potentially influencing factor mass estimates and loadings, and in turn, the associations between source-specific mass estimates and outcomes. A lack of monitoring data for gases and/or carbonaceous species also raise the possibility that the source categories identified in our study are serving as surrogates for other unmeasured correlated constituents actually responsible for the observed adverse health effects.

## 4. Conclusions

This is the first study to have partitioned cardiometabolic health effects of PM pollution to specific sources of PM in a Middle Eastern city, suggesting that the toxicity of PM did vary by composition. PM_2.5_ exposure increased the prevalence of MetS, hyperglycemia and hypertension. PM_2.5_ trace elements from soil/road dust, such as Al and Si, were significantly associated with hyperglycemia and hypertension. Also, a significant association between PM_2.5_ traffic source components (Pb, S) and hyperglycemia was observed. Ni content in PM might be responsible for the observed effects. This may help decision makers to take strict measures towards limiting exposure to specified components like Ni for example. A larger and more robust study with greater spatial variability for pollutants and detailed adjustments for ecological confounding could enable a more definitive evaluation of the PM-MetS relationship.

## Figures and Tables

**Table 1:** Study cohort characteristics by location.

		Total	Al-Nuzlah	Al-Alfiyya	Al-Rehab	Pitrumin	Al-Rughama	University	Al-Muhammadiyah
		N = 2025	N = 266	N = 157	N = 409	N = 104	N = 103	N = 697	N = 298
Age	Mean (SD)	31.46 (10.69)	29.43 (11.36)	35.26 (11.73)	32.28 (10.41)	30.66 (10.80)	28.38 (8.73)	31.37 (10.98)	31.75 (9.06)
Sex	Male N (%)	996 (49.2)	185 (70.1)	130 (86.1)	175 (42.8)	42 (40.4)	61 (59.2)	246 (35.3)	157 (52.9)
	Female N (%)	1029 (50.8)	79 (29.9)	21 (13.9)	234 (57.2)	62 (59.6)	42 (40.8)	451 (64.7)	140 (47.1)
Smoking	Non-smoker N (%)	1493 (73.7)	219 (82.9)	103 (68.2)	296 (72.4)	80 (76.9)	59 (57.3)	526 (75.5)	210 (70.7)
	Smoker N (%)	532 (26.3)	45 (17.0)	48 (31.8)	113 (27.6)	24 (23.1)	44 (42.7)	171 (24.5)	87 (29.3)
Fruit and Vegetable Intake	≤4 times/week N (%)	959 (47.4)	164 (62.1)	88 (58.3)	143 (35.0)	46 (44.2)	49 (476.)	342 (49.1)	127 (42.8)
	5–6 times/week N (%)	787 (38.9)	82 (31.1)	55 (36.4)	170 (41.6)	49 (47.1)	39 (37.9)	276 (39.6)	116 (39.1)
	≥7 times/week N (%)	279 (13.8)	18 (6.8)	9 (5.3)	96 (23.5)	9 (8.6)	15 (14.6)	79 (11.3)	54 (18.2)
Residence	House N (%)	681 (40.0)	152 (57.6)	25 (16.6)	92 (22.5)	74 (71.2)	58 (56.3)	221 (31.7)	59 (19.9)
	Apartment N (%)	1261 (68.9)	108 (40.9)	87 (57.6)	301 (73.6)	30 (28.8)	44 (42.7)	467 (67.0)	224 (75.4)
	Villa N (%)	83 (3.1)	4 (1.5)	39 (25.8)	16 (3.9)	0 (0.0)	1 (0.1)	9 (1.3)	14 (4.7)
Marietal Status	No N (%)	971 (48.0)	164 (62.1)	44 (29.1)	163 (39.9)	57 (54.8)	63 (61.2)	351 (50.3)	129 (43.4)
	Yes N (%)	1054 (52.0)	100 (37.9)	107 (70.9)	246 (60.1)	47 (45.2)	40 (38.8)	346 (49.6)	168 (56.6)
Type of Work	No Work N (%)	523 (25.8)	73 (27.7)	30 (19.9)	146 (35.7)	32 (30.8)	23 (22.3)	129 (18.5)	90 (30.3)
	Labor Work N (%)	1339 (66.1)	160 (60.6)	76 (50.3)	223 (54.5)	69 (66.3)	80 (77.7)	562 (80.6)	169 (56.9)
	Desk Work N (%)	163 (8.1)	31 (11.7)	45 (29.8)	40 (9.8)	3 (2.9)	0 (0.0)	6 (0.9)	38 (12.8)
Education Level	Illiterate N (%)	67 (3.3)	17 (6.4)	2 (1.3)	15 (3.7)	3 (2.9)	2 (1.9)	28 (4.0)	0 (0.0)
	Can Read & Write N (%)	59 (2.9)	8 (3.0)	2 (1.3)	11 (2.7)	3 (2.9)	0 (0.0)	26 (3.7)	9 (3.0)
	Primary School N (%)	30 (1.5)	9 (3.5)	2 (1.3)	3 (0.7)	6 (5.8)	0 (0.0)	8 (1.1)	2 (0.7)
	Prepatory School N (%)	116 (5.7)	33 (12.5)	1 (0.7)	23 (5.6)	10 (9.6)	5 (4.9)	31 (4.5)	13 (4.4)
	Secondary School N (%)	758 (37.5)	109 (41.3)	52 (34.4)	172 (42.1)	43 (41.3)	38 (36.9)	230 (32.9)	114 (38.4)
	University Degree N (%)	969 (47.9)	85 (32.2)	90 (59.6)	174 (42.5)	38 (36.5)	57 (55.3)	369 (52.9)	156 (52.5)
	Post Graduate N (%)	26 (1.3)	3 (1.1)	2 (1.3)	11 (2.7)	1 (1.0)	1 (1.0)	5 (0.7)	3 (1.0)
Physical Activity	No N (%)	1315 (64.9)	143 (64.2)	37 (24.5)	323 (78.9)	56 (53.8)	54 (52.5)	496 (71.2)	206 (69.4)
	Yes N (%)	710 (35.1)	121 (45.8)	114 (75.5)	86 (21.0)	48 (46.2)	49 (47.5)	201 (28.8)	91 (30.6)
Metabolic Syndrome	No N (%)	1656 (81.8)	232 (87.9)	133 (88.1)	290 (70.9)	86 (82.7)	91 (88.3)	607 (87.1)	217 (73.1)
	Yes N (%)	369 (18.2)	32 (12.1)	18 (11.9)	119 (29.1)	18 (17.3)	12 (11.7)	90 (12.9)	80 (26.9)
Hyperglycemia	No N (%)	941 (46.5)	186 (70.5)	122 (80.8)	215 (52.6)	77 (74.0)	74 (71.8)	491 (70.4)	158 (53.2)
	Yes N (%)	1084 (53.5)	78 (29.5)	29 (19.2)	194 (47.4)	27 (26.0)	29 (28.2)	206 (29.6)	139 (46.8)
Hypertension	No N (%)	1323 (65.3)	132 (50.0)	86 (56.9)	147 (35.9)	45 (43.3)	45 (43.7)	357 (51.2)	129 (43.4)
	Yes N (%)	702 (34.7)	132 (50.0)	65 (43.1)	262 (64.1)	59 (56.7)	58 (56.3)	340 (48.8)	168 (56.6)
Obesity	No N (%)	659 (32.5)	112 (42.4)	64 (42.4)	129 (31.5)	29 (27.9)	41 (39.8)	226 (32.4)	58 (19.5)
	Yes N (%)	1366 (67.5)	152 (57.6)	87 (57.6)	280 (68.5)	75 (72.1)	62 (60.2)	471 (67.6)	239 (80.5)

**Table 2:** PM and elemental constituents concentration levels (Mean ± S.D., μg/m^3^) by location.

	**Total**		**Al-Nuzlah**		**Al-Alfiyya**		**Al-Rehab**	
	**PM_2.5_**	**PM_10_**	**PM_2.5_**	**PM_10_**	**PM_2.5_**	**PM_10_**	**PM_2.5_**	**PM_10_**
PM	29.07 (18.68)	85.12 (30.30)	29.10 (14.11)	73.51 (10.13)	24.51 (11.77)	99.42 (43.94)	18.04 (3.96)	68.95 (20.48)
Al	0.89 (1.35)	3.42 (2.05)	0.28 (0.066)	2.06 (0.46)	0.58 (0.36)	4.79 (2.50)	0.28 (0.12)	2.41 (0.86)
Ca	0.63 (0.77)	4.18 (1.33)	0.31 (0.037)	3.55 (0.49)	0.36 (0.18)	4.48 (2.35)	0.28 (0.18)	3.48 (1.32)
Cr	0.0028 (0.0031)	0.0093 (0.0058)	0.0018 (0.00077)	0.0066 (0.0011)	0.0021 (0.00087)	0.013 (0.0073)	0.0014 (0.00099)	0.0059 (0.0023)
Cu	0.0061 (0.0041)	0.017 (0.0076)	0.0024 (0.0023)	0.012 (0.0048)	0.013 (0.0097)	0.022 (0.015)	0.0018 (0.0014)	0.013 (0.0038)
Fe	0.72 (1.18)	3.32 (2.24)	0.17 (0.027)	1.84 (0.38)	0.37 (0.21)	4.25 (2.43)	0.23 (0.15)	2.29 (0.86)
K	0.21 (0.17)	0.72 (0.27)	0.15 (0.033)	0.52 (0.083)	0.013 (0.063)	0.86 (0.51)	0.11 (0.030)	0.56 (0.19)
Mg	0.33 (0.43)	1.44 (0.52)	0.20 (0.079)	1.17 (0.16)	0.23 (0.15)	1.93 (0.87)	0.11 (0.057)	1.21 (0.54)
Mn	0.022 (0.033)	0.10 (0.063)	0.0054 (0.0018)	0.053 (0.012)	0.012 (0.0057)	0.12 (0.062)	0.009 (0.0048)	0.069 (0.028)
Ni	0.0071 (0.0028)	0.011 (0.0039)	0.0071 (0.0023)	0.099 (0.0027)	0.0064 (0.0033)	0.012 (0.0067)	0.0042 (0.00091)	0.0073 (0.0025)
Pb	0.13 (0.14)	0.16 (0.17)	0.037 (0.049)	0.039 (0.044)	0.49 (0.44)	0.47 (0.47)	0.086 (0.14)	0.11 (0.15)
S	3.48 (0.75)	3.39 (0.51)	4.04 (0.45)	3.76 (0.47)	3.87 (2.34)	3.47 (2.17)	2.51 (0.35)	2.69 (0.15)
Si	2.47 (3.99)	11.19 (6.70)	0.72 (0.23)	7.08 (1.36)	1.44 (0.89)	15.29 (8.10)	0.68 (0.43)	7.69 (2.80)
Sr	0.0049 (0.0061)	0.026 (0.0085)	0.0021 (0.00066)	0.022 (0.0034)	0.003 (0.0019)	0.029 (0.015)	0.0018 (0.0020)	0.023 (0.015)
Ti	0.070 (0.13)	0.32 (0.25)	0.011 (0.0027)	0.15 (0.028)	0.032 (0.021)	0.42 (0.26)	0.017 (0.014)	0.21 (0.08)
V	0.025 (0.010)	0.032 (0.012)	0.029 (0.0098)	0.33 (0.0096)	0.023 (0.012)	0.031 (0.015)	0.015 (0.0038)	0.021 (0.06)
Zn	0.038 (0.023)	0.073 (0.044)	0.017 (0.0069)	0.039 (0.012)	0.056 (0.064)	0.13 (0.17)	0.019 (0.0088)	0.049 (0.011)
	**Pitrumin**		**Al-Rughama**		**University**		**Al-Muhammadiyah**	
	**PM_2.5_**	**PM_10_**	**PM_2.5_**	**PM_10_**	**PM_2.5_**	**PM_10_**	**PM_2.5_**	**PM_10_**
PM	31.14 (5.85)	107.13 (28.75)	73.16 (65.08)	141.27 (124.20)	29.91 (11.67)	85.67 (33.09)	15.78 (3.05)	47.01 (6.52)
Al	0.41 (0.22)	3.51 (1.07)	3.93 (4.07)	7.17 (6.07)	0.63 (1.02)	3.07 (1.77)	0.15 (0.43)	0.91 (0.41)
Ca	0.53 (0.17)	6.11 (1.42)	2.36 (2.76)	4.95 (2.94)	0.39 (0.30)	4.77 (2.18)	0.19 (0.15)	1.96 (0.61)
Cr	0.0015 (0.0010)	0.0089 (0.0031)	0.0097 (0.010)	0.021 (0.020)	0.0019 (0.0021)	0.0083 (0.0053)	0.00092 (0.00058)	0.0022 (0.00081)
Cu	0.0054 (0.0053)	0.029 (0.0096)	0.0098 (0.0057)	0.016 (0.011)	0.0077 (0.011)	0.019 (0.016)	0.0029 (0.0037)	0.0048 (0.0022)
Fe	0.36 (0.15)	3.59 (1.16)	3.37 (3.94)	7.71 (7.73)	0.39 (0.64)	2.81 (1.59)	0.12 (0.093)	0.75 (0.22)
K	0.21 (0.091)	0.91 (0.29)	0.59 (0.62)	1.14 (0.93)	0.16 (0.13)	0.69 (0.31)	0.12 (0.024)	0.35 (0.075)
Mg	0.19 (0.098)	1.55 (0.47)	1.29 (1.21)	2.21 (1.51)	0.25 (0.33)	1.41 (0.60)	0.058 (0.025)	1.55 (0.16)
Mn	0.014 (0.0073)	0.11 (0.035)	0.097 (0.11)	0.23 (0.21)	0.015 (0.021)	0.095 (0.052)	0.0051 (0.0028)	0.11 (0.0079)
Ni	0.012 (0.0048)	0.018 (0.0044)	0.0097 (0.0058)	0.13 (0.0088)	0.0058 (0.0022)	0.011 (0.0044)	0.0043 (0.00096)	0.018 (0.0021)
Pb	0.031 (0.020)	0.041 (0.022)	0.17 (0.27)	0.021 (0.34)	0.21 (0.42)	0.27 (0.52)	0.0066 (0.0067)	0.0093 (0.0063)
S	4.51 (0.97)	4.06 (0.67)	3.65 (1.32)	2.82 (1.25)	3.11 (1.19)	3.71 (0.99)	2.63 (0.47)	3.19 (0.73)
Si	1.16 (0.62)	12.18 (3.96)	11.49 (12.57)	23.35 (20.07)	1.47 (2.46)	10.07 (5.42)	0.34 (0.21)	2.64 (1.04)
Sr	0.0044 (0.0017)	0.036 (0.021)	0.019 (0.022)	0.036 (0.025)	0.0029 (0.0027)	0.027 (0.014)	0.0021 (0.00097)	0.012 (0.003)
Ti	0.025 (0.014)	0.31 (0.095)	0.36 (0.43)	0.83 (0.90)	0.034 (0.069)	0.25 (0.17)	0.012 (0.0094)	0.07 (0.03)
V	0.045 (0.0099)	0.055 (0.0072)	0.028 (0.015)	0.031 (0.014)	0.019 (0.0069)	0.029 (0.0099)	0.016 (0.0037)	0.021 (0.0078)
Zn	0.073 (0.13)	0.13 (0.17)	0.043 (0.027)	0.061 (0.036)	0.044 (0.0.76)	0.079 (0.13)	0.0099 (0.0044)	0.022 (0.005)

**Table 3:** Risk Ratios (RR) and 95% Confidence Interval (CI) per 10 μg/m^3^ increment of particulate matter (PM) and factor mass estimates.

	Metabolic Syndrome	Hyperglycemia	Hypertension	BMI
PM_2.5_	1.11 (1.05–1.18)	1.08 (1.03–1.14)	1.09 (1.04–1.14)	0.95 (0.91–1.00)
Factor 1: Soil & Road Dust	1.16 (1.08–1.25)	1.12 (1.06–1.19)	1.11 (1.05–1.18)	0.95 (0.90–1.01)
Factor 2: Residual Oil	0.94 (0.88–1.00)	0.88 (0.77–1.01)	0.98 (0.96–1.01)	1.00 (0.98–1.03)
Factor 3: Incineration	0.99 (0.92–1.07)	0.98 (0.91–1.05)	1.00 (0.96–1.04)	0.85 (0.76–0.95)
Factor 4: Traffic	1.28 (0.94–1.76)	1.33 (1.05–1.71)	1.12 (0.89–1.12)	0.74 (0.55–1.00)
PM_10_	1.03 (0.95–1.13)	0.98 (0.92–1.05)	1.06 (1.00–1.13)	0.95 (0.89–1.02)
Factor 1: Soil & Road Dust	1.03 (0.95–1.10)	0.99 (0.95–1.05)	1.05 (1.00–1.10)	0.99 (0.89–1.10)
Factor 2: Incineration	1.02 (0.82–1.26)	0.90 (0.76–1.06)	1.08 (0.93–1.26)	0.84 (0.61–1.17)
Factor 3: Traffic	1.01 (0.56–1.83)	1.02 (0.67–1.57)	0.77 (0.53–1.12)	1.13 (0.90–1.40)
